# Crystal structure of [(2*S*)-1-[(3*S*)-3-carboxy-6,7-dimethoxy-1,2,3,4-tetra­hydroisoquinolin-2-yl]-1-oxopropan-2-yl][(2*S*)-1-ethoxy-1-oxo-4-phenylbutan-2-yl]azanium chloride acetonitrile monosolvate

**DOI:** 10.1107/S160053681402090X

**Published:** 2014-09-24

**Authors:** Ai-zhen Li, Wen-jie Xu

**Affiliations:** a Shenzhen Salubris Pharmaceuticals CO., Ltd, Guangdong Shenzhen 361021, People’s Republic of China

**Keywords:** crystal structure, moexipril hydro­chloride, hydrogen bond

## Abstract

The title compound (trivial name moexipril hydro­chloride) crystallizes as the aceto­nitrile monosolvate, C_27_H_35_N_2_O_7_^+^·Cl^−^·C_2_H_3_N, with the salt comprising a U-shaped cation and a chloride anion. The conformation of the cation is stabilized by a weak intra­molecular N^+^—H⋯O hydrogen bond and the tetra­hydro­pyridine ring adopts a *boat* conformation. The dihedral angle between the planes of the benzene rings is 85.6 (1)°. In the crystal, the cations and anions form tight ionic pairs by strong inter­molecular O—H⋯Cl hydrogen bonds. C—H⋯Cl and C—H⋯N hydrogen bonds link these ionic pairs and the aceto­nitrile solvate mol­ecules into puckered layers parallel to (100).

## Related literature   

For the synthesis of the title compound, see: Klutchko *et al.* (1986 ▶); Yamazaki & Suzuki (1998 ▶). For the structure and applications of related compounds, see: Suzuki *et al.* (2000 ▶, 2010 ▶). 
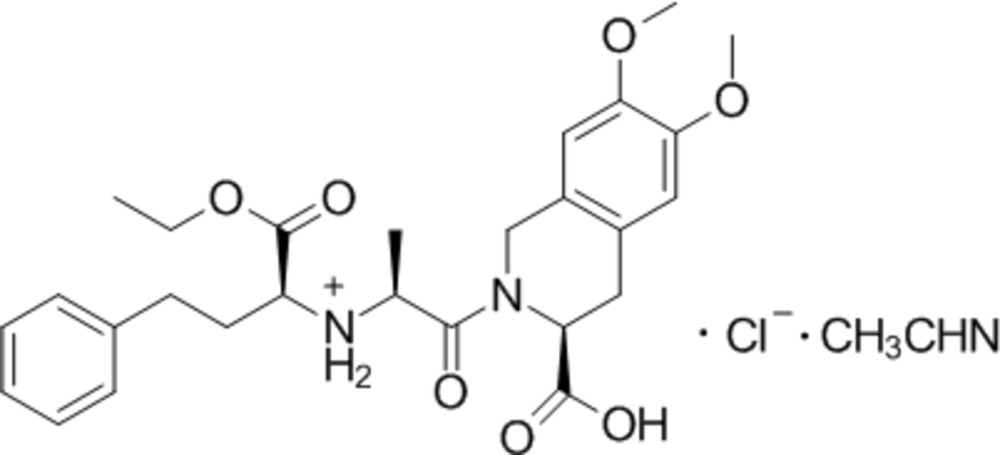


## Experimental   

### Crystal data  

C_27_H_35_N_2_O_7_^+^·Cl^−^·C_2_H_3_N
*M* *_r_* = 576.07
Monoclinic, 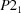

*a* = 10.9391 (6) Å
*b* = 10.4655 (4) Å
*c* = 13.3159 (5) Å
β = 97.419 (4)°
*V* = 1511.68 (12) Å^3^
*Z* = 2
Cu *K*α radiation
μ = 1.52 mm^−1^
*T* = 103 K
0.50 × 0.20 × 0.20 mm

### Data collection  

Agilent Xcalibur, Eos, Gemini diffractometer
Absorption correction: multi-scan (*CrysAlis PRO*; Agilent, 2011 ▶) *T*_min_ = 0.516, *T*_max_ = 0.750
11078 measured reflections
5728 independent reflections
5570 reflections with *I* > 2σ(*I*)
*R*_int_ = 0.021

### Refinement  

*R*[*F* ^2^ > 2σ(*F* ^2^)] = 0.033
*wR*(*F* ^2^) = 0.087
*S* = 1.03
5728 reflections
367 parameters
1 restraint
H-atom parameters constrained
Δρ_max_ = 0.55 e Å^−3^
Δρ_min_ = −0.34 e Å^−3^
Absolute structure: Flack (1983 ▶), 2620 Friedel pairs
Absolute structure parameter: 0.000 (10)

### 

Data collection: *CrysAlis PRO* (Agilent, 2011 ▶); cell refinement: *CrysAlis PRO*; data reduction: *CrysAlis PRO*; program(s) used to solve structure: *SHELXTL* (Sheldrick, 2008 ▶); program(s) used to refine structure: *SHELXTL*; molecular graphics: *SHELXTL*; software used to prepare material for publication: *SHELXTL*.

## Supplementary Material

Crystal structure: contains datablock(s) I, global. DOI: 10.1107/S160053681402090X/kq2014sup1.cif

Structure factors: contains datablock(s) I. DOI: 10.1107/S160053681402090X/kq2014Isup2.hkl

Supporting information file. DOI: 10.1107/S160053681402090X/kq2014Isup3.tif

Supporting information file. DOI: 10.1107/S160053681402090X/kq2014Isup4.tif

Supporting information file. DOI: 10.1107/S160053681402090X/kq2014Isup5.cml

**Fig. 1.**
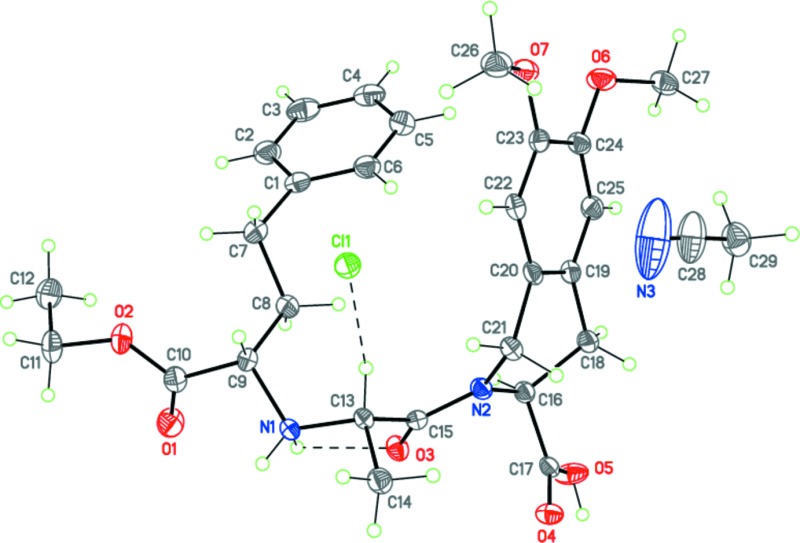
Molecular structure of the title compound. Displacement ellipsoids are presented at the 50% probability level. H atoms are depicted as small spheres of arbitrary radius. Dashed lines indicate the intramolecular N—H···O and the intermolecular C—H···Cl hydrogen bonds.

. DOI: 10.1107/S160053681402090X/kq2014fig1.tif
Mol­ecular structure of the title compound. Displacement ellipsoids are presented at the 50% probability level. H atoms are depicted as small spheres of arbitrary radius. Dashed lines indicate the intra­molecular N—H⋯O and the inter­molecular C—H⋯Cl hydrogen bonds.

**Fig. 2.**
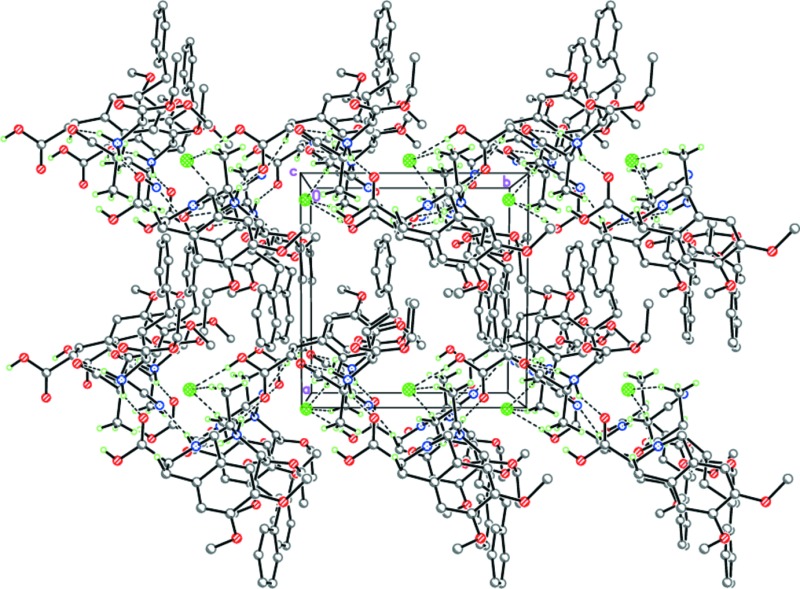
Crystal packing showing the puckered layers parallel to (100). Dashed lines indicate the intra- and intermolecular hydrogen bonds.

. DOI: 10.1107/S160053681402090X/kq2014fig2.tif
Crystal packing showing the puckered layers parallel to (100). Dashed lines indicate the intra- and inter­molecular hydrogen bonds.

CCDC reference: 1024892

Additional supporting information:  crystallographic information; 3D view; checkCIF report

## Figures and Tables

**Table 1:** Hydrogen-bond geometry (Å, °)

*D*—H⋯*A*	*D*—H	H⋯*A*	*D*⋯*A*	*D*—H⋯*A*
O5—H5⋯Cl1^i^	0.84	2.09	2.924 (3)	171
N1—H1*A*⋯O3	0.92	2.04	2.587 (2)	117
N1—H1*B*⋯O4^ii^	0.92	1.87	2.724 (3)	154
C13—H13⋯Cl1	1.00	2.52	3.459 (2)	157
C14—H14*B*⋯Cl1^iii^	0.98	2.71	3.593 (2)	150
C18—H18*A*⋯N3^iv^	0.99	2.39	3.367 (4)	168
C29—H29*A*⋯Cl1^iv^	0.98	2.74	3.698 (2)	165

Symmetry codes: (i) 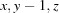; (ii) 
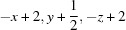
; (iii) 
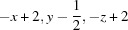
; (iv) 
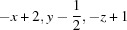
.

## supplementary crystallographic information

### S1. Comment

Moexipril is a nonsulfhydryl containing precursor of the active angiotensin-converting enzyme. It is well known that the polymorphic and *pseudo*-polymorphic crystals of moexipril show different physico-chemical properties (Klutchko *et al.*, 1986; Suzuki *et al.*, 2010). The crystal structure of the β-form, the monohydrate and the sesquihydrate have been already reported previously (Yamazaki & Suzuki, 1998; Suzuki *et al.*, 2000). In this paper, we report the *X*-ray crystal structure and stereochemistry of moexipril hydrochloride acetonitrile monosolvate.

### S2. Experimental

A solution of 2-[2-[(1-Ethoxycarbonyl-3-phenylpropyl)amino]propanoyl]-6,7-dimethoxy–3,4-dihydro-1*H*-isoquinoline-3-carboxylic acid in absolute ethanol was treated with hydrochloride in the presence of 5% Pd/C. The resulting mixture was concentrated and stood for 3 h at reduced pressure. The residue obtained was recrystallized from acetonitrile at room temperature to give colourless crystals of I suitable for X-ray diffraction analysis.

### S3. Refinement

The absolute structure of I was objectively determined by the refinement of Flack parameter (2620 (94%) Friedel pairs measured), which has become equal to 0.00 (1). The calculated Hooft parameter is equal to -0.003 (6).

The hydroxyl and amino hydrogen atoms were objectively localized in the difference-Fourier map and include into refinement with fixed positional and isotropic displacement parameters [*U_i_*_so_(H) = 1.5*U*_eq_(O) and *U_i_*_so_(H) = 1.2*U*_eq_(N)]. The other hydrogen atoms were placed in the calculated positions with C—H distances = 0.95 (aryl-H), 0.98 (methyl-H), 0.99 (methylene-H) and 1.00 (methine-H) Å and refined in the riding model with fixed isotropic displacement parameters [*U_i_*_so_(H) = 1.5*U*_eq_(C) for the methyl groups and 1.2*U*_eq_(C) for the other groups].

### Crystal data

**Table:**

C_27_H_35_N_2_O_7_^+^·Cl^−^·C_2_H_3_N	*F*(000) = 612
*M**_r_* = 576.07	*D*_x_ = 1.266 Mg m^−^^3^
Monoclinic, *P*2_1_	Cu *K*α radiation, λ = 1.54184 Å
Hall symbol: P 2yb	Cell parameters from 6099 reflections
*a* = 10.9391 (6) Å	θ = 3.3–71.7°
*b* = 10.4655 (4) Å	µ = 1.52 mm^−^^1^
*c* = 13.3159 (5) Å	*T* = 103 K
β = 97.419 (4)°	Block, colourless
*V* = 1511.68 (12) Å^3^	0.50 × 0.20 × 0.20 mm
*Z* = 2	

### Data collection

**Table:**

Agilent Xcalibur, Eos, Gemini diffractometer	5728 independent reflections
Radiation source: fine-focus sealed tube	5570 reflections with *I* > 2σ(*I*)
Graphite monochromator	*R*_int_ = 0.021
Detector resolution: 16.0971 pixels mm^-1^	θ_max_ = 71.8°, θ_min_ = 3.4°
ω scans	*h* = −13→12
Absorption correction: multi-scan (*CrysAlis PRO*; Agilent, 2011)	*k* = −12→12
*T*_min_ = 0.516, *T*_max_ = 0.750	*l* = −16→16
11078 measured reflections	

### Refinement

**Table:**

Refinement on *F*^2^	Secondary atom site location: difference Fourier map
Least-squares matrix: full	Hydrogen site location: difference Fourier map
*R*[*F*^2^ > 2σ(*F*^2^)] = 0.033	H-atom parameters constrained
*wR*(*F*^2^) = 0.087	*w* = 1/[σ^2^(*F*_o_^2^) + (0.0513*P*)^2^ + 0.3525*P*] where *P* = (*F*_o_^2^ + 2*F*_c_^2^)/3
*S* = 1.03	(Δ/σ)_max_ = 0.001
5728 reflections	Δρ_max_ = 0.55 e Å^−^^3^
367 parameters	Δρ_min_ = −0.34 e Å^−^^3^
1 restraint	Absolute structure: Flack (1983), 2620 Friedel pairs
Primary atom site location: structure-invariant direct methods	Absolute structure parameter: 0.000 (10)

### Special details

**Table:**

Geometry. All e.s.d.'s (except the e.s.d. in the dihedral angle between two l.s. planes) are estimated using the full covariance matrix. The cell e.s.d.'s are taken into account individually in the estimation of e.s.d.'s in distances, angles and torsion angles; correlations between e.s.d.'s in cell parameters are only used when they are defined by crystal symmetry. An approximate (isotropic) treatment of cell e.s.d.'s is used for estimating e.s.d.'s involving l.s. planes.
Refinement. Refinement of *F*^2^ against ALL reflections. The weighted *R*-factor *wR* and goodness of fit *S* are based on *F*^2^, conventional *R*-factors *R* are based on *F*, with *F* set to zero for negative *F*^2^. The threshold expression of *F*^2^ > σ(*F*^2^) is used only for calculating *R*-factors(gt) *etc*. and is not relevant to the choice of reflections for refinement. *R*-factors based on *F*^2^ are statistically about twice as large as those based on *F*, and *R*-factors based on ALL data will be even larger.

### Fractional atomic coordinates and isotropic or equivalent isotropic displacement parameters (Å^2^)

**Table:**

	*x*	*y*	*z*	*U*_iso_*/*U*_eq_	
Cl1	0.93335 (4)	0.48179 (4)	0.82049 (3)	0.02184 (10)	
O6	0.52273 (13)	0.26128 (14)	0.40381 (11)	0.0274 (3)	
O5	0.79427 (13)	−0.28384 (13)	0.76776 (12)	0.0286 (3)	
H5	0.8374	−0.3472	0.7887	0.043*	
O3	0.81278 (12)	−0.00568 (13)	0.94514 (9)	0.0204 (3)	
O4	0.96943 (12)	−0.17651 (13)	0.81353 (10)	0.0214 (3)	
O2	0.73833 (13)	0.45383 (13)	1.15813 (10)	0.0240 (3)	
C22	0.77885 (16)	0.30103 (17)	0.60435 (13)	0.0174 (3)	
H22	0.8293	0.3680	0.6349	0.021*	
O1	0.73521 (14)	0.24231 (14)	1.19090 (11)	0.0285 (3)	
O7	0.65710 (12)	0.44801 (12)	0.48979 (10)	0.0229 (3)	
N2	0.85280 (14)	0.05279 (14)	0.78967 (11)	0.0173 (3)	
C25	0.62888 (17)	0.10402 (18)	0.51823 (14)	0.0204 (4)	
H25	0.5763	0.0373	0.4902	0.024*	
C17	0.86058 (16)	−0.17996 (17)	0.78125 (12)	0.0170 (3)	
C23	0.68475 (16)	0.32815 (18)	0.52690 (13)	0.0185 (3)	
C15	0.86015 (16)	0.06883 (17)	0.88977 (13)	0.0168 (3)	
C26	0.7471 (2)	0.54471 (19)	0.51712 (15)	0.0278 (4)	
H26A	0.7196	0.6252	0.4840	0.042*	
H26B	0.8257	0.5190	0.4954	0.042*	
H26C	0.7578	0.5564	0.5908	0.042*	
N1	0.87847 (13)	0.20668 (14)	1.03592 (10)	0.0154 (3)	
H1A	0.8400	0.1330	1.0528	0.018*	
H1B	0.9429	0.2227	1.0859	0.018*	
C20	0.79909 (16)	0.17574 (18)	0.63698 (12)	0.0172 (3)	
C6	0.50803 (18)	0.34869 (19)	0.75184 (15)	0.0242 (4)	
H6	0.5901	0.3387	0.7369	0.029*	
C8	0.67413 (17)	0.28867 (19)	0.95447 (14)	0.0209 (4)	
H8A	0.7007	0.2557	0.8911	0.025*	
H8B	0.6235	0.2219	0.9818	0.025*	
C24	0.61083 (17)	0.22814 (19)	0.48234 (14)	0.0202 (4)	
C13	0.92855 (16)	0.18674 (17)	0.93702 (12)	0.0160 (3)	
H13	0.9074	0.2624	0.8923	0.019*	
C21	0.90294 (16)	0.13908 (17)	0.71794 (13)	0.0174 (3)	
H21A	0.9693	0.0957	0.6870	0.021*	
H21B	0.9379	0.2164	0.7536	0.021*	
C14	1.06858 (16)	0.17006 (19)	0.95410 (13)	0.0207 (4)	
H14A	1.0983	0.1501	0.8896	0.031*	
H14B	1.0900	0.1001	1.0020	0.031*	
H14C	1.1071	0.2493	0.9817	0.031*	
C9	0.78851 (16)	0.31546 (17)	1.03144 (13)	0.0171 (3)	
H9	0.8295	0.3954	1.0118	0.021*	
C18	0.75488 (17)	−0.05633 (18)	0.63247 (13)	0.0202 (4)	
H18A	0.8264	−0.0883	0.6013	0.024*	
H18B	0.6840	−0.1132	0.6111	0.024*	
C16	0.78556 (16)	−0.06097 (17)	0.74921 (13)	0.0170 (3)	
H16	0.7063	−0.0656	0.7790	0.020*	
C12	0.5522 (2)	0.4949 (2)	1.23442 (16)	0.0331 (5)	
H12A	0.5295	0.5680	1.1899	0.050*	
H12B	0.5200	0.5075	1.2990	0.050*	
H12C	0.5170	0.4166	1.2021	0.050*	
C2	0.36654 (19)	0.3986 (2)	0.86908 (16)	0.0288 (4)	
H2	0.3510	0.4224	0.9351	0.035*	
C10	0.75218 (16)	0.33115 (19)	1.13720 (14)	0.0203 (4)	
C19	0.72420 (17)	0.07729 (18)	0.59540 (13)	0.0185 (4)	
C28	0.9733 (2)	0.2135 (3)	0.4377 (2)	0.0452 (6)	
C29	0.9077 (2)	0.1088 (2)	0.38715 (17)	0.0317 (5)	
H29A	0.9422	0.0895	0.3245	0.048*	
H29B	0.8205	0.1315	0.3709	0.048*	
H29C	0.9155	0.0335	0.4313	0.048*	
C27	0.49224 (18)	0.1638 (2)	0.32940 (14)	0.0260 (4)	
H27A	0.4375	0.1993	0.2721	0.039*	
H27B	0.4506	0.0933	0.3595	0.039*	
H27C	0.5678	0.1322	0.3056	0.039*	
C1	0.48732 (18)	0.38403 (19)	0.84900 (15)	0.0235 (4)	
C11	0.69039 (18)	0.4832 (2)	1.25307 (14)	0.0283 (4)	
H11A	0.7266	0.5643	1.2812	0.034*	
H11B	0.7138	0.4145	1.3030	0.034*	
C3	0.26836 (19)	0.3788 (2)	0.79368 (18)	0.0326 (5)	
H3	0.1862	0.3895	0.8082	0.039*	
C5	0.41007 (19)	0.3281 (2)	0.67694 (15)	0.0276 (4)	
H5A	0.4253	0.3031	0.6111	0.033*	
C4	0.28987 (19)	0.3435 (2)	0.69729 (17)	0.0303 (4)	
H4	0.2228	0.3300	0.6455	0.036*	
N3	1.0276 (3)	0.2938 (3)	0.4810 (3)	0.0866 (12)	
C7	0.59528 (19)	0.40899 (19)	0.93067 (15)	0.0268 (4)	
H7A	0.5636	0.4384	0.9931	0.032*	
H7B	0.6473	0.4778	0.9079	0.032*	

### Atomic displacement parameters (Å^2^)

**Table:**

	*U*^11^	*U*^22^	*U*^33^	*U*^12^	*U*^13^	*U*^23^
Cl1	0.0299 (2)	0.01598 (18)	0.01952 (19)	0.00160 (16)	0.00259 (15)	0.00102 (15)
O6	0.0268 (7)	0.0263 (7)	0.0257 (7)	0.0052 (6)	−0.0092 (6)	0.0006 (6)
O5	0.0232 (7)	0.0160 (7)	0.0432 (9)	−0.0014 (5)	−0.0095 (6)	0.0032 (6)
O3	0.0274 (6)	0.0178 (6)	0.0161 (5)	−0.0040 (5)	0.0039 (5)	0.0010 (5)
O4	0.0188 (6)	0.0201 (6)	0.0235 (7)	0.0006 (5)	−0.0033 (5)	0.0023 (5)
O2	0.0287 (7)	0.0232 (7)	0.0213 (6)	−0.0008 (5)	0.0084 (5)	−0.0059 (5)
C22	0.0185 (8)	0.0208 (9)	0.0135 (8)	−0.0032 (7)	0.0040 (6)	−0.0023 (6)
O1	0.0358 (8)	0.0269 (7)	0.0250 (7)	0.0003 (6)	0.0121 (6)	0.0033 (6)
O7	0.0258 (7)	0.0203 (7)	0.0211 (6)	0.0029 (5)	−0.0020 (5)	0.0027 (5)
N2	0.0198 (7)	0.0162 (7)	0.0150 (7)	−0.0004 (6)	−0.0006 (6)	−0.0003 (6)
C25	0.0200 (9)	0.0220 (9)	0.0184 (8)	−0.0002 (7)	−0.0005 (7)	−0.0030 (7)
C17	0.0204 (8)	0.0181 (9)	0.0117 (8)	0.0003 (7)	−0.0007 (6)	−0.0003 (6)
C23	0.0204 (8)	0.0206 (9)	0.0156 (8)	0.0040 (7)	0.0068 (6)	0.0016 (7)
C15	0.0179 (8)	0.0163 (8)	0.0157 (8)	0.0040 (6)	0.0009 (6)	0.0003 (6)
C26	0.0367 (11)	0.0193 (10)	0.0260 (10)	0.0000 (8)	−0.0019 (8)	0.0028 (8)
N1	0.0168 (7)	0.0175 (7)	0.0116 (6)	0.0005 (5)	0.0006 (5)	−0.0003 (5)
C20	0.0172 (8)	0.0235 (9)	0.0112 (7)	0.0007 (7)	0.0037 (6)	0.0000 (7)
C6	0.0213 (9)	0.0263 (10)	0.0253 (10)	0.0046 (7)	0.0037 (7)	0.0044 (8)
C8	0.0204 (9)	0.0230 (9)	0.0187 (9)	0.0022 (7)	−0.0002 (7)	−0.0019 (7)
C24	0.0169 (8)	0.0270 (10)	0.0162 (8)	0.0036 (7)	−0.0003 (7)	0.0006 (7)
C13	0.0197 (8)	0.0167 (8)	0.0116 (7)	0.0018 (7)	0.0017 (6)	0.0007 (6)
C21	0.0173 (8)	0.0206 (9)	0.0142 (8)	−0.0013 (6)	0.0015 (6)	0.0003 (6)
C14	0.0190 (9)	0.0254 (10)	0.0179 (8)	0.0000 (7)	0.0026 (7)	−0.0005 (7)
C9	0.0189 (8)	0.0155 (8)	0.0172 (8)	0.0010 (7)	0.0030 (6)	−0.0025 (6)
C18	0.0243 (9)	0.0191 (9)	0.0160 (8)	0.0011 (7)	−0.0015 (7)	−0.0027 (7)
C16	0.0188 (8)	0.0170 (8)	0.0151 (8)	−0.0006 (7)	0.0010 (6)	−0.0008 (6)
C12	0.0349 (11)	0.0371 (12)	0.0291 (10)	0.0074 (10)	0.0111 (8)	−0.0039 (9)
C2	0.0284 (10)	0.0317 (11)	0.0274 (10)	0.0094 (8)	0.0079 (8)	0.0054 (8)
C10	0.0158 (8)	0.0251 (9)	0.0203 (9)	−0.0016 (7)	0.0032 (7)	−0.0035 (7)
C19	0.0200 (9)	0.0210 (9)	0.0146 (8)	0.0016 (7)	0.0029 (7)	−0.0018 (7)
C28	0.0409 (13)	0.0531 (16)	0.0477 (14)	−0.0144 (12)	0.0293 (12)	−0.0160 (12)
C29	0.0277 (10)	0.0404 (12)	0.0278 (10)	−0.0020 (9)	0.0064 (8)	−0.0019 (9)
C27	0.0229 (9)	0.0316 (10)	0.0207 (9)	−0.0006 (8)	−0.0071 (7)	0.0004 (8)
C1	0.0251 (9)	0.0203 (9)	0.0248 (9)	0.0070 (7)	0.0020 (8)	0.0032 (7)
C11	0.0311 (10)	0.0326 (10)	0.0222 (9)	−0.0015 (9)	0.0077 (7)	−0.0106 (9)
C3	0.0211 (10)	0.0326 (11)	0.0443 (13)	0.0066 (8)	0.0054 (9)	0.0093 (9)
C5	0.0323 (10)	0.0259 (10)	0.0239 (10)	0.0029 (8)	0.0013 (8)	0.0012 (8)
C4	0.0261 (10)	0.0230 (10)	0.0388 (12)	0.0017 (8)	−0.0069 (8)	0.0042 (8)
N3	0.081 (2)	0.088 (2)	0.105 (2)	−0.0498 (18)	0.0661 (19)	−0.059 (2)
C7	0.0289 (10)	0.0260 (11)	0.0243 (9)	0.0101 (8)	−0.0004 (8)	−0.0035 (7)

### Geometric parameters (Å, º)

**Table:**

O6—C24	1.372 (2)	C13—C14	1.529 (2)
O6—C27	1.431 (2)	C13—H13	1.0000
O5—C17	1.306 (2)	C21—H21A	0.9900
O5—H5	0.8400	C21—H21B	0.9900
O3—C15	1.232 (2)	C14—H14A	0.9800
O4—C17	1.213 (2)	C14—H14B	0.9800
O2—C10	1.327 (2)	C14—H14C	0.9800
O2—C11	1.462 (2)	C9—C10	1.521 (2)
C22—C23	1.389 (2)	C9—H9	1.0000
C22—C20	1.390 (3)	C18—C19	1.506 (3)
C22—H22	0.9500	C18—C16	1.548 (2)
O1—C10	1.202 (2)	C18—H18A	0.9900
O7—C23	1.368 (2)	C18—H18B	0.9900
O7—C26	1.426 (2)	C16—H16	1.0000
N2—C15	1.336 (2)	C12—C11	1.505 (3)
N2—C16	1.465 (2)	C12—H12A	0.9800
N2—C21	1.471 (2)	C12—H12B	0.9800
C25—C24	1.389 (3)	C12—H12C	0.9800
C25—C19	1.395 (3)	C2—C3	1.388 (3)
C25—H25	0.9500	C2—C1	1.390 (3)
C17—C16	1.522 (2)	C2—H2	0.9500
C23—C24	1.406 (3)	C28—N3	1.141 (4)
C15—C13	1.535 (2)	C28—C29	1.430 (3)
C26—H26A	0.9800	C29—H29A	0.9800
C26—H26B	0.9800	C29—H29B	0.9800
C26—H26C	0.9800	C29—H29C	0.9800
N1—C9	1.501 (2)	C27—H27A	0.9800
N1—C13	1.505 (2)	C27—H27B	0.9800
N1—H1A	0.9200	C27—H27C	0.9800
N1—H1B	0.9200	C1—C7	1.521 (3)
C20—C19	1.386 (3)	C11—H11A	0.9900
C20—C21	1.511 (2)	C11—H11B	0.9900
C6—C5	1.383 (3)	C3—C4	1.385 (3)
C6—C1	1.392 (3)	C3—H3	0.9500
C6—H6	0.9500	C5—C4	1.386 (3)
C8—C7	1.536 (2)	C5—H5A	0.9500
C8—C9	1.537 (2)	C4—H4	0.9500
C8—H8A	0.9900	C7—H7A	0.9900
C8—H8B	0.9900	C7—H7B	0.9900
			
C24—O6—C27	115.19 (15)	C10—C9—C8	110.46 (14)
C17—O5—H5	109.5	N1—C9—H9	109.4
C10—O2—C11	116.64 (15)	C10—C9—H9	109.4
C23—C22—C20	119.84 (16)	C8—C9—H9	109.4
C23—C22—H22	120.1	C19—C18—C16	111.62 (14)
C20—C22—H22	120.1	C19—C18—H18A	109.3
C23—O7—C26	116.47 (14)	C16—C18—H18A	109.3
C15—N2—C16	115.59 (14)	C19—C18—H18B	109.3
C15—N2—C21	126.25 (15)	C16—C18—H18B	109.3
C16—N2—C21	118.14 (14)	H18A—C18—H18B	108.0
C24—C25—C19	120.04 (17)	N2—C16—C17	109.56 (14)
C24—C25—H25	120.0	N2—C16—C18	112.16 (14)
C19—C25—H25	120.0	C17—C16—C18	110.26 (14)
O4—C17—O5	125.15 (16)	N2—C16—H16	108.2
O4—C17—C16	122.96 (16)	C17—C16—H16	108.2
O5—C17—C16	111.88 (14)	C18—C16—H16	108.2
O7—C23—C22	124.31 (17)	C11—C12—H12A	109.5
O7—C23—C24	116.23 (16)	C11—C12—H12B	109.5
C22—C23—C24	119.46 (17)	H12A—C12—H12B	109.5
O3—C15—N2	122.80 (17)	C11—C12—H12C	109.5
O3—C15—C13	118.93 (15)	H12A—C12—H12C	109.5
N2—C15—C13	118.26 (15)	H12B—C12—H12C	109.5
O7—C26—H26A	109.5	C3—C2—C1	120.7 (2)
O7—C26—H26B	109.5	C3—C2—H2	119.7
H26A—C26—H26B	109.5	C1—C2—H2	119.7
O7—C26—H26C	109.5	O1—C10—O2	126.37 (17)
H26A—C26—H26C	109.5	O1—C10—C9	123.12 (18)
H26B—C26—H26C	109.5	O2—C10—C9	110.47 (16)
C9—N1—C13	112.41 (13)	C20—C19—C25	119.49 (17)
C9—N1—H1A	109.1	C20—C19—C18	117.64 (16)
C13—N1—H1A	109.1	C25—C19—C18	122.78 (16)
C9—N1—H1B	109.1	N3—C28—C29	177.4 (4)
C13—N1—H1B	109.1	C28—C29—H29A	109.5
H1A—N1—H1B	107.9	C28—C29—H29B	109.5
C19—C20—C22	120.94 (16)	H29A—C29—H29B	109.5
C19—C20—C21	116.57 (16)	C28—C29—H29C	109.5
C22—C20—C21	122.49 (16)	H29A—C29—H29C	109.5
C5—C6—C1	120.51 (18)	H29B—C29—H29C	109.5
C5—C6—H6	119.7	O6—C27—H27A	109.5
C1—C6—H6	119.7	O6—C27—H27B	109.5
C7—C8—C9	112.03 (15)	H27A—C27—H27B	109.5
C7—C8—H8A	109.2	O6—C27—H27C	109.5
C9—C8—H8A	109.2	H27A—C27—H27C	109.5
C7—C8—H8B	109.2	H27B—C27—H27C	109.5
C9—C8—H8B	109.2	C2—C1—C6	118.74 (18)
H8A—C8—H8B	107.9	C2—C1—C7	120.87 (18)
O6—C24—C25	123.58 (17)	C6—C1—C7	120.37 (17)
O6—C24—C23	116.26 (16)	O2—C11—C12	109.82 (15)
C25—C24—C23	120.15 (16)	O2—C11—H11A	109.7
N1—C13—C14	110.89 (13)	C12—C11—H11A	109.7
N1—C13—C15	104.89 (14)	O2—C11—H11B	109.7
C14—C13—C15	113.26 (15)	C12—C11—H11B	109.7
N1—C13—H13	109.2	H11A—C11—H11B	108.2
C14—C13—H13	109.2	C4—C3—C2	120.15 (19)
C15—C13—H13	109.2	C4—C3—H3	119.9
N2—C21—C20	108.06 (14)	C2—C3—H3	119.9
N2—C21—H21A	110.1	C6—C5—C4	120.46 (19)
C20—C21—H21A	110.1	C6—C5—H5A	119.8
N2—C21—H21B	110.1	C4—C5—H5A	119.8
C20—C21—H21B	110.1	C3—C4—C5	119.44 (19)
H21A—C21—H21B	108.4	C3—C4—H4	120.3
C13—C14—H14A	109.5	C5—C4—H4	120.3
C13—C14—H14B	109.5	C1—C7—C8	111.73 (16)
H14A—C14—H14B	109.5	C1—C7—H7A	109.3
C13—C14—H14C	109.5	C8—C7—H7A	109.3
H14A—C14—H14C	109.5	C1—C7—H7B	109.3
H14B—C14—H14C	109.5	C8—C7—H7B	109.3
N1—C9—C10	107.06 (14)	H7A—C7—H7B	107.9
N1—C9—C8	111.17 (14)		
			
C26—O7—C23—C22	14.3 (2)	C15—N2—C16—C18	166.61 (15)
C26—O7—C23—C24	−166.07 (16)	C21—N2—C16—C18	−12.1 (2)
C20—C22—C23—O7	179.68 (16)	O4—C17—C16—N2	−16.0 (2)
C20—C22—C23—C24	0.0 (3)	O5—C17—C16—N2	165.23 (15)
C16—N2—C15—O3	−0.8 (2)	O4—C17—C16—C18	107.90 (19)
C21—N2—C15—O3	177.83 (16)	O5—C17—C16—C18	−70.85 (19)
C16—N2—C15—C13	−179.54 (14)	C19—C18—C16—N2	−36.4 (2)
C21—N2—C15—C13	−0.9 (3)	C19—C18—C16—C17	−158.82 (15)
C23—C22—C20—C19	−2.0 (3)	C11—O2—C10—O1	−4.1 (3)
C23—C22—C20—C21	177.53 (15)	C11—O2—C10—C9	173.57 (14)
C27—O6—C24—C25	−29.9 (3)	N1—C9—C10—O1	−39.3 (2)
C27—O6—C24—C23	150.20 (17)	C8—C9—C10—O1	81.9 (2)
C19—C25—C24—O6	177.08 (17)	N1—C9—C10—O2	142.99 (15)
C19—C25—C24—C23	−3.0 (3)	C8—C9—C10—O2	−95.82 (17)
O7—C23—C24—O6	2.7 (2)	C22—C20—C19—C25	1.4 (3)
C22—C23—C24—O6	−177.65 (16)	C21—C20—C19—C25	−178.13 (16)
O7—C23—C24—C25	−177.23 (16)	C22—C20—C19—C18	178.01 (16)
C22—C23—C24—C25	2.5 (3)	C21—C20—C19—C18	−1.5 (2)
C9—N1—C13—C14	133.60 (15)	C24—C25—C19—C20	1.1 (3)
C9—N1—C13—C15	−103.80 (15)	C24—C25—C19—C18	−175.32 (17)
O3—C15—C13—N1	−21.2 (2)	C16—C18—C19—C20	44.4 (2)
N2—C15—C13—N1	157.68 (15)	C16—C18—C19—C25	−139.15 (18)
O3—C15—C13—C14	99.90 (19)	C3—C2—C1—C6	−0.2 (3)
N2—C15—C13—C14	−81.27 (19)	C3—C2—C1—C7	178.4 (2)
C15—N2—C21—C20	−124.98 (18)	C5—C6—C1—C2	−0.3 (3)
C16—N2—C21—C20	53.6 (2)	C5—C6—C1—C7	−178.89 (19)
C19—C20—C21—N2	−46.4 (2)	C10—O2—C11—C12	−92.1 (2)
C22—C20—C21—N2	134.08 (17)	C1—C2—C3—C4	0.3 (3)
C13—N1—C9—C10	−176.13 (14)	C1—C6—C5—C4	0.7 (3)
C13—N1—C9—C8	63.13 (18)	C2—C3—C4—C5	0.1 (3)
C7—C8—C9—N1	−167.02 (15)	C6—C5—C4—C3	−0.6 (3)
C7—C8—C9—C10	74.3 (2)	C2—C1—C7—C8	118.7 (2)
C15—N2—C16—C17	−70.60 (18)	C6—C1—C7—C8	−62.7 (2)
C21—N2—C16—C17	110.70 (16)	C9—C8—C7—C1	175.96 (16)

### Hydrogen-bond geometry (Å, º)

**Table:**

*D*—H···*A*	*D*—H	H···*A*	*D*···*A*	*D*—H···*A*
O5—H5···Cl1^i^	0.84	2.09	2.924 (3)	171
N1—H1*A*···O3	0.92	2.04	2.587 (2)	117
N1—H1*B*···O4^ii^	0.92	1.87	2.724 (3)	154
C13—H13···Cl1	1.00	2.52	3.459 (2)	157
C14—H14*B*···Cl1^iii^	0.98	2.71	3.593 (2)	150
C18—H18*A*···N3^iv^	0.99	2.39	3.367 (4)	168
C29—H29*A*···Cl1^iv^	0.98	2.74	3.698 (2)	165

Symmetry codes: (i) *x*, *y*−1, *z*; (ii) −*x*+2, *y*+1/2, −*z*+2; (iii) −*x*+2, *y*−1/2, −*z*+2; (iv) −*x*+2, *y*−1/2, −*z*+1.
