# Supplementary figures and images for: Crystal structure of [(2S)-1-[(3S)-3-carboxy-6,7-dimethoxy-1,2,3,4-tetra­hydroisoquinolin-2-yl]-1-oxopropan-2-yl][(2S)-1-ethoxy-1-oxo-4-phenylbutan-2-yl]azanium chloride acetonitrile monosolvate
Source: Acta Crystallogr Sect E Struct Rep Online. 2014 Sep 24;70(Pt 10):o1126–7. doi: 10.1107/S160053681402090X (PMC4257232; doi:10.1107/S160053681402090X)

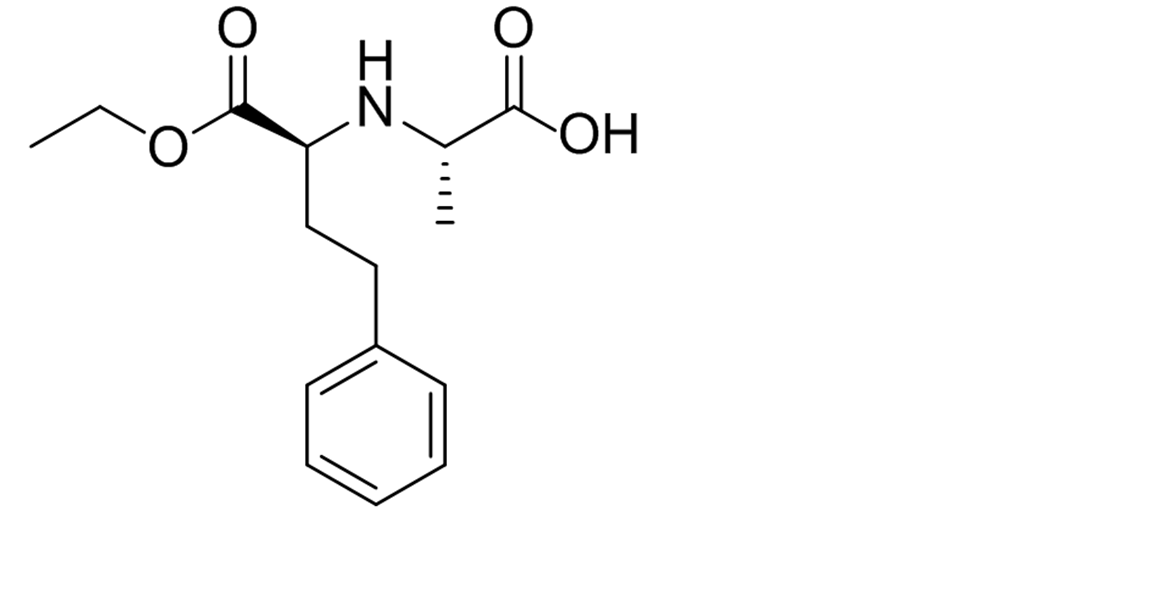

Supplement: Supplementary file 3 [file e-70-o1126-Isup3.tif]

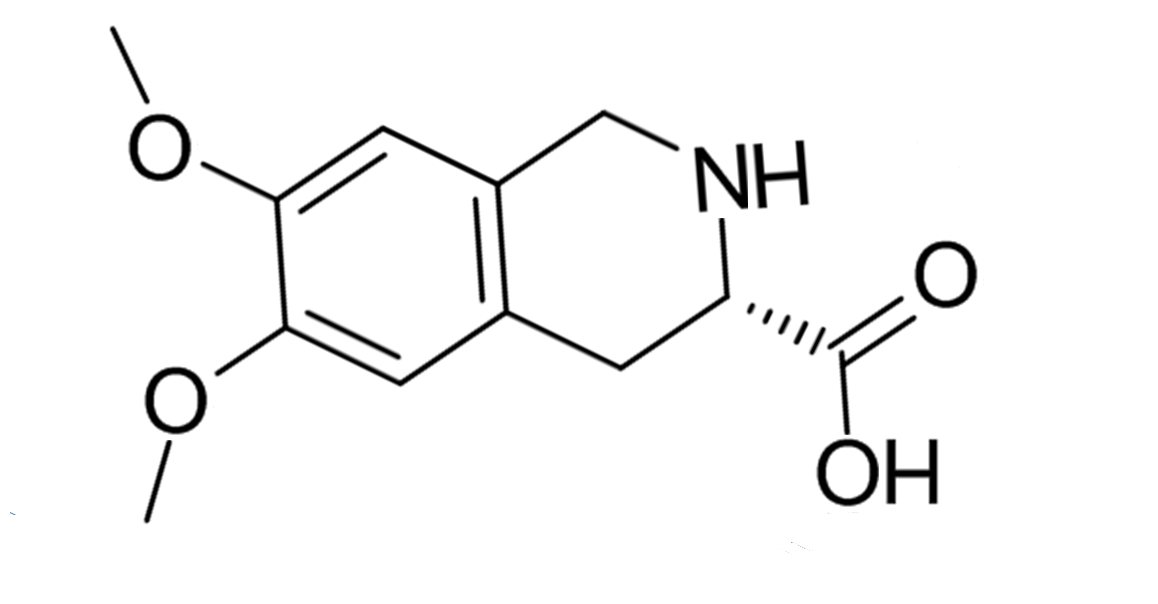

Supplement: Supplementary file 4 [file e-70-o1126-Isup4.tif]

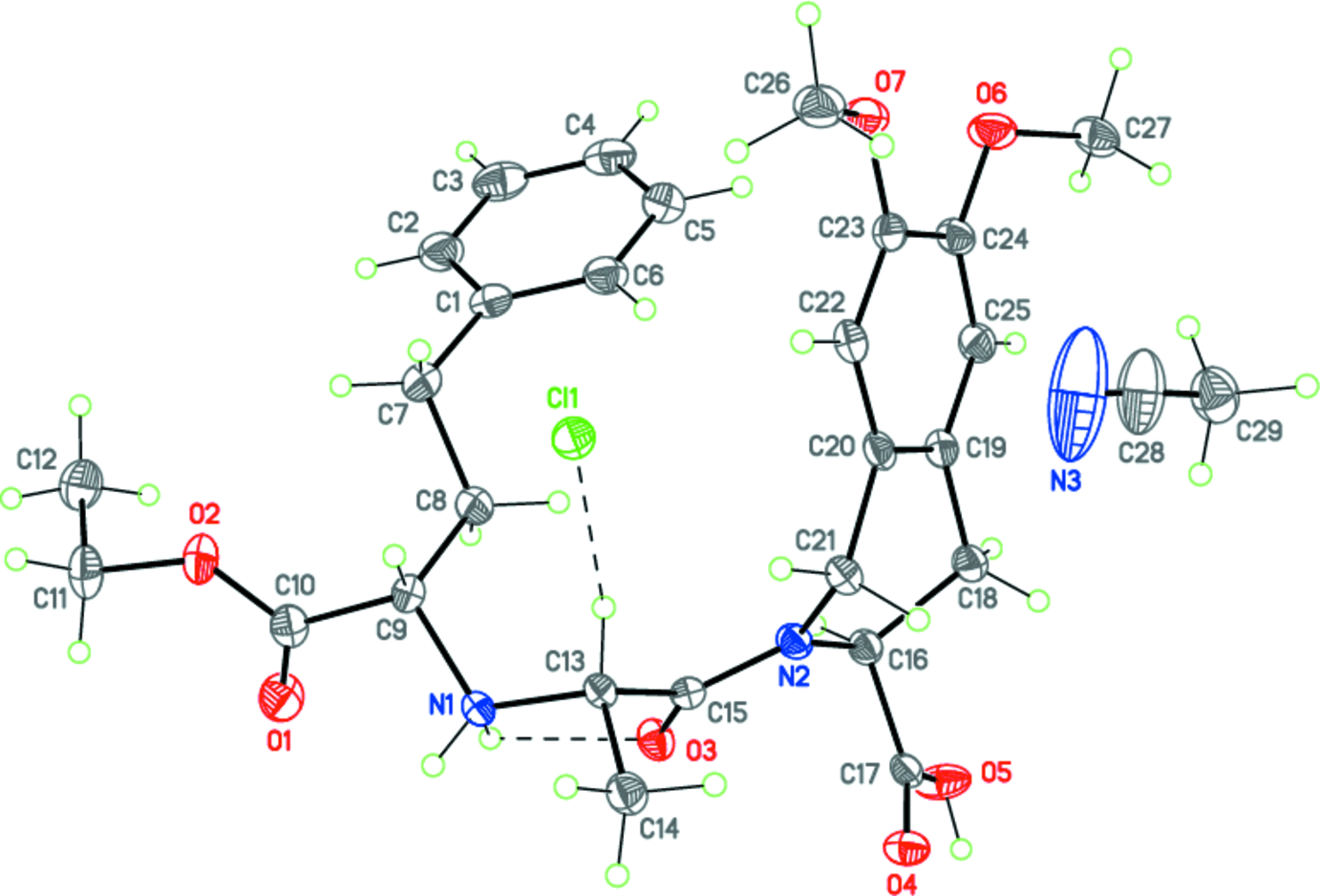

Supplement: Supplementary file 6 [file e-70-o1126-fig1.tif]

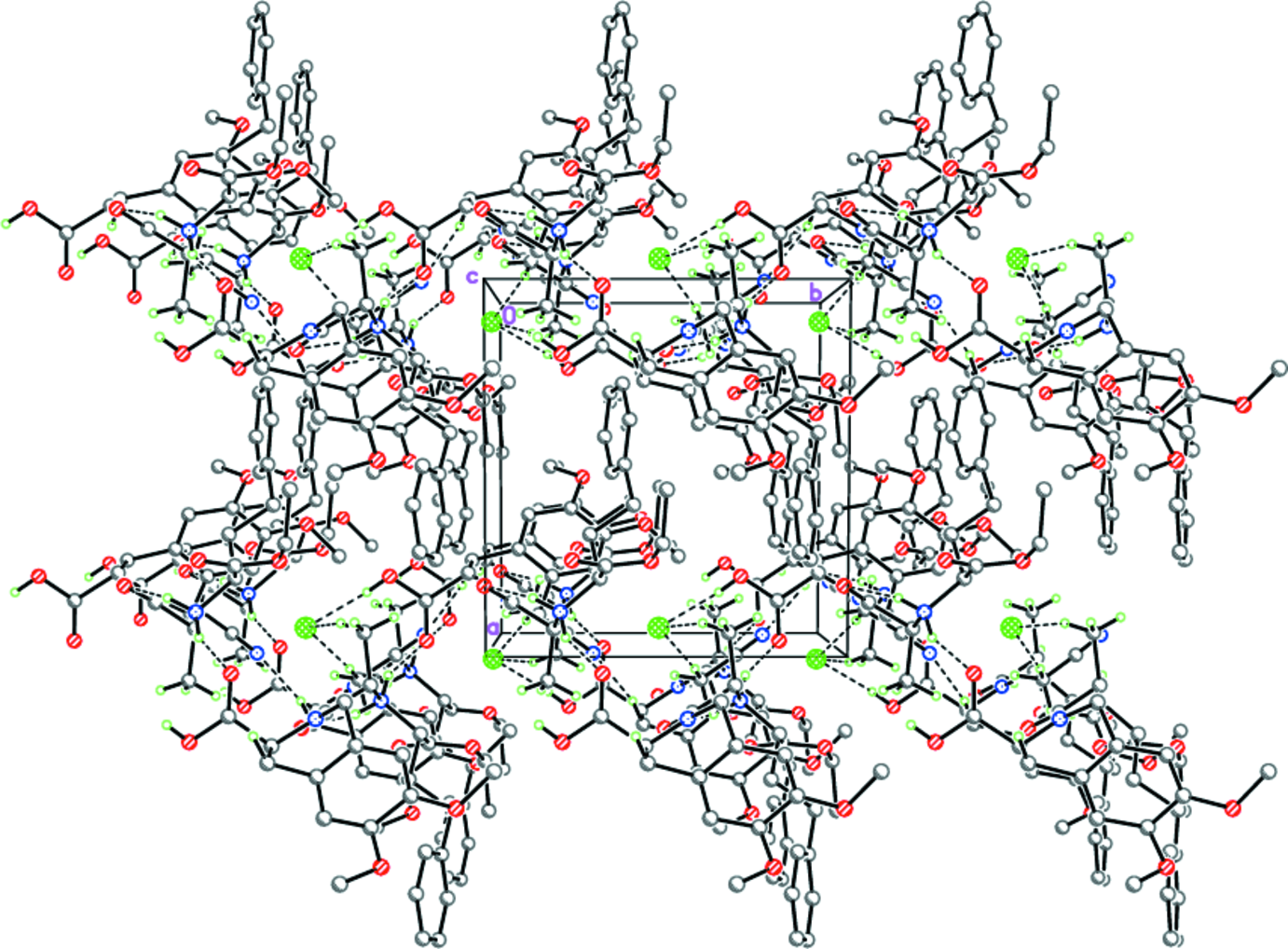

Supplement: Supplementary file 7 [file e-70-o1126-fig2.tif]
